# Supplementary material for: Widespread and intron-rich mirusviruses are predicted to reproduce in nuclei of unicellular eukaryotes
Source: Nat Microbiol. 2025 Nov 28;11(1):228–39. doi: 10.1038/s41564-025-02190-6 (PMC12768974; doi:10.1038/s41564-025-02190-6)
Supplement: Supplementary file 1 — Supplementary Figs. 1–3 and discussion. [file 41564_2025_2190_MOESM1_ESM.pdf]

nature microbiology

Supplementary information https://doi.org/10.1038/s41564-025-02190-6

Widespread and intron-rich mirusviruses are predicted to reproduce in nuclei of unicellular eukaryotes

In the format provided by the authors and unedited

Genome-resolved metagenomics applied to *Mirusviricota*

- **Two distinct genome-resolved metagenomic surveys for *Mirusviricota*:**

Our global genome-resolved metagenomic analyses (a.k.a, binning) are delineated into two distinct and complementary surveys, which we briefly summarize here before detailing them more extensively in dedicated sections.

First, we performed **manual binning and curation of large *Tara* Oceans metagenomic co-assemblies with the bioinformatic platform anvi'o**[1,2], using the mirusvirus MCP as guidance among previously processed metabins[3] (see https://anvio.org/blog/mirus-discovery/ and https://anvio.org/blog/constrained-binning/ for details about the metabins and our "single marker gene" binning strategy). This manual binning and curation effort allowed a first genomic characterization of a wide range of *Mirusviricota* lineages, with high confidence in their biological relevance.

Second, we performed an **iterative automated binning of thousands of mOTUs bins containing at last one mirusvirus MCP, using a framework we developed for this study**, which considerably expanded the scope of our genomic exploration for *Mirusviricota*. Notably, the third iteration characterized most of the cryptic MAGs.

Overall, both surveys relied heavily on the mirusvirus MCP to guide the binning. Manual binning provided limited number of genomes but was critical to the success of the automated binning, as it provided the first genomes for major clades of mirusviruses that allowed identification of a broad range of *Mirusviricota* contigs.

- **Manual binning and curation with anvi'o:**

Mirusviruses were first discovered[4] by performing a phylogeny-guided genome-resolved metagenomic approach focused on the RNApolB marker gene and applied to large *Tara* Oceans metagenomic co-assemblies[3] (https://anvio.org/blog/mirus-discovery/ for details). This was the first round of mirusvirus manual binning and curation with anvi'o. Metagenome-assembled genomes (MAGs) corresponding to the putative order *Demutovirales* were characterized and analysed with Alphafold[5] to identify core genes corresponding to the mirusvirus major capsid protein (MCP)[4].

In the present study, we improved the Hidden Markov model (HMM) for mirusvirus MCPs and used this HMM to perform a second round of mirusvirus manual binning and curation with anvi'o, still within the same large *Tara* Oceans metagenomic co-assemblies. This second round allowed the recovery of mirusvirus MAGs far beyond the limited evolutionary scope of *Demutovirales*. We gained high confidence in the biological relevance and overall quality of those MAGs based on multiple criteria. First, MAGs contain contigs that are highly congruent in terms of distribution across *Tara* Oceans metagenomes (differential main coverage information) and sequence composition (the broadly used tetranucleotide frequency), following state-of-the-art

binning techniques. Second, manual inspection of each MAG (and if needs be the removal of contigs) provided a critical gain of confidence. Third, we build preliminary single copy core gene collections for major clades of mirusviruses (mainly within the scope of *Demutovirales*, *Okeanovirales* and *Styxvirales*), allowing estimation of the completion and redundancy of most of MAGs. Such approach is relatively common in the field of viral genomics and was already applied to previously characterized lineages of mirusviruses using a slightly different strategy[6]. Completion and redundancy estimates are common practice for bacterial, archaeal and eukaryotic MAGs. We and others found that those estimates work equally well for large and giant eukaryotic viruses.

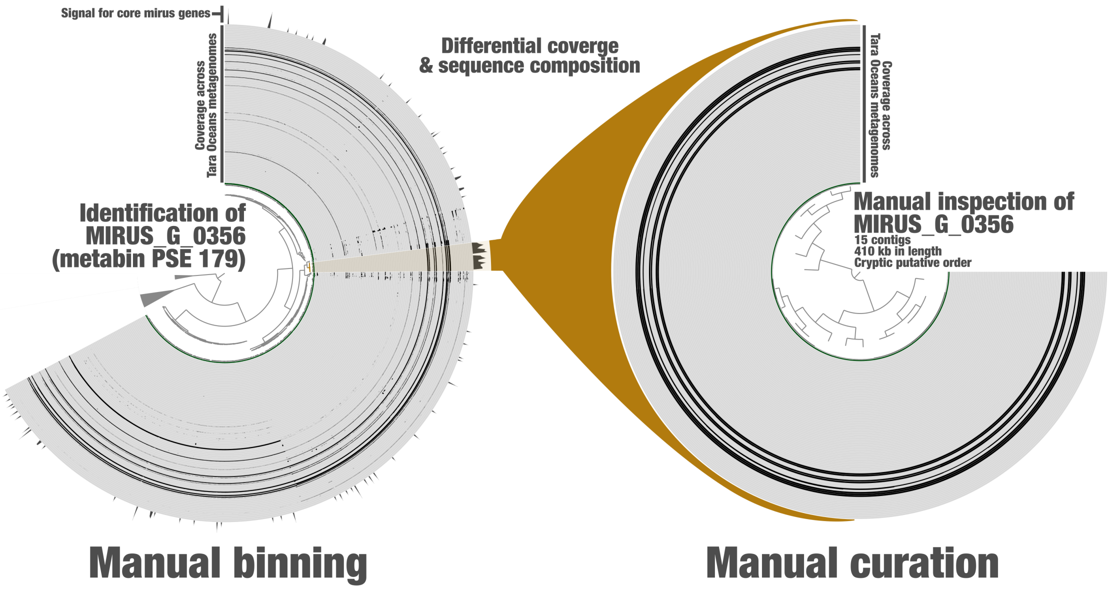

Figure 1: Identification of the MIRUS_G_0356 MAG from the *Tara* Oceans metabin PSE_179 corresponding to a subset of Pacific Southeast large metagenomic co-assembly with the interactive interface of anvi'o. Contigs from the metabin are clustered based on both differential coverage across *Tara* Oceans metagenomes and sequence composition (left panel). Mean coverage of contigs is displayed as individual layers for each metagenome. The outer layer displays an overall signal for dozens of mirusvirus core genes identified with an HMM collection within anvi'o. The selected contigs correspond to the MAG labelled as "MIRUS_G_0356" in our final non-redundant genomic database. Right panel shows the manual inspection of the "MIRUS_G_0356" contigs alone, using the same displays and clustering strategy. In this example, no curation was needed given the highly coherent signal of all contigs.

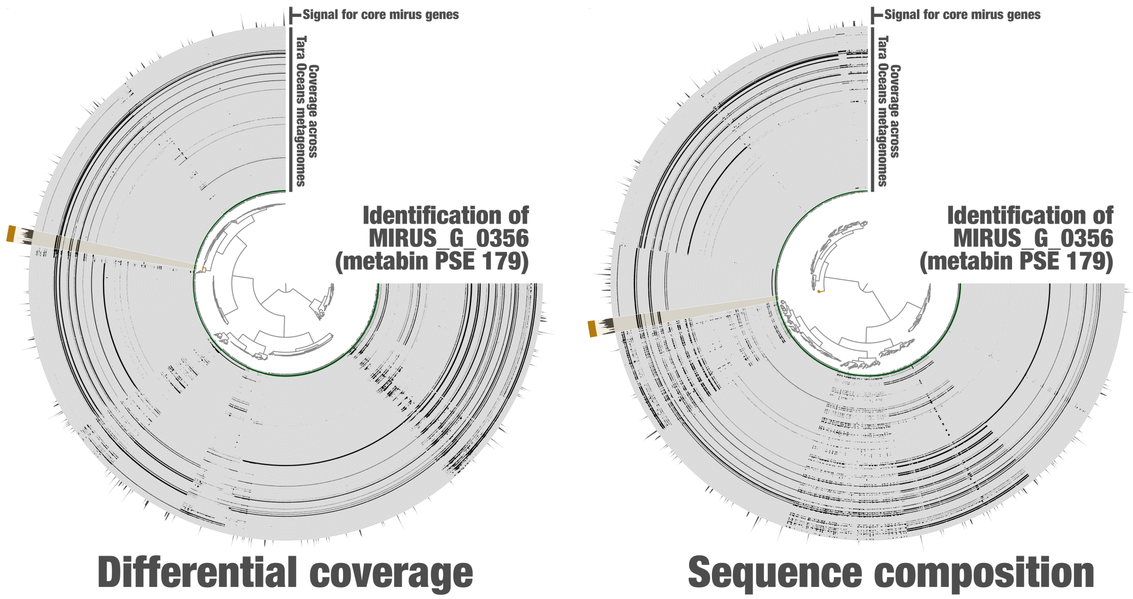

Figure 2: Clustering of contigs in the metabin PSE_179 using differential coverage alone (left panel) or sequence composition alone (right panel). In both panels, the selected contigs correspond to "MIRUS_G_0356". Displays in the anvi'o interactive interface are the same as those presented in the Figure 1.

Critically, the 115 MAGs characterized from this second round of manual binning and those characterized from the first round (n=111) cover all the main families of *Demutovirales*, *Okeanovirales* and *Styxvirales*. The MAGs also cover few of the cryptic putative orders, for which we provide an example that (1) explains the main principles of mirusvirus manual binning and curation in the anvi'o interactive interface (Figure 1), (2) and showcases the relevance of both differential coverage alone and sequence composition alone for binning large and giant eukaryotic virus genomes, similar to what is already well documented within the scope of *Tara* Oceans for Bacteria/Archaea[7,8] and Eukarya[3].

In the final non-redundant database of 1,257 mirusvirus genomes described in our study, just 63 genomes correspond to MAGs characterized from this second round of mirusvirus manual binning and curation with anvi'o. Yet, this binning was instrumental to our study. It revealed a much wider evolutionary diversity for *Mirusviricota* (initially restrained to *Demutovirales*) and allowed the characterization of MAGs that are highly trusted thanks to their manual inspection in the anvi'o interactive interface. However, manual binning and curation is a slow and labour-intensive process that simply cannot be applied to the vast and ever-growing legacy of metagenomic data sets. To embrace this metagenomic legacy at a global scale, we transitioned from the manual binning of few metagenomic co-assemblies processed within anvi'o to an iterative automated binning effort applied to tens of thousands of metagenomic assemblies built within the scope of the mOTUs database[9,10].

- **Iterative automated binning of the mOTUs database:**

mOTUs bins: The mOTUs database[9,10] provides access to many metagenomic assemblies from a wide range of ecosystems (generated with MetaSPAdes[11]), along with automatically generated bins (binned with metabat2[12] using differential coverage and sequence composition) and their genes (predicted with prodigal[13]). Here, we ran the improved HMM for mirusvirus MCPs against the entire pool of proteins from mOTUs (version from May 2024) and recovered 2,659 bins containing at least one mirusvirus MCP. After removing contigs <2.5 kb in length, those 2,659 bins contained a total of 2,1 million contigs for a cumulative length of 5.96 Gb. Those 2.1 million contigs were analysed in the context of individual bins to characterize mirusvirus MAGs using the strategy outlined below.

A global reference genomic database: Our iterative automated binning strategy relies heavily on contig-level taxonomic inferences for decontamination purposes. As a result, our approach (which is labelled as an "automated binning" due to the use of metabat2 bins from mOTUs as a starting point) might be better described as an "automatic decontamination of bins". Indeed, our goal was to identify as many mirusvirus contigs as possible from those bins, and to exclude the remaining contigs prior to defining individual mirusvirus MAGs. To best decontaminate those bins, we created a comprehensive database of more than 5,000 genomes (primarily MAGs from our 10 years of binning with *Tara* Oceans) that covers a broad range of mainly marine lineages corresponding to Bacteria/Archaea[7,8], eukaryotes[3] (including their chloroplasts[14]), *Mirusviricota*/*Nucleocytoviricota* (GOEV resource[4]), as well as an extended database of mirusviruses corresponding to (1) few genomes from the literature[15–17], (2) MAGs characterized from the second round of mirusvirus manual binning and curation with anvi'o, (3) and mOTUs contigs >100 kb in length and containing a mirusvirus MCP. We created a reference database of proteins from this database and labelled each protein to a high-ranking taxonomy (Bacteria, Archaea, Eukarya, chloroplast, *Mirusviricota, Nucleocytoviricota*). We also labelled each mirusvirus protein to a preliminary *Mirusviricota* clade, with most of them being labelled to the equivalent of *Demutovirales*, *Okeanovirales* or *Styxvirales* (formal naming was done much later in our investigation).

Decontamination of the global reference genomic database: We learned during the early development of our iterative automated binning workflow that few genomes from the reference genomic database had low to medium levels of contamination from a distant taxonomic source (e.g., some *Mirusviricota* contigs occurred in a few bacterial MAGs characterized before the discovery of mirusviruses). Critically, we found that even a very low volume of contaminations could have some dramatic consequences when inferring the high-ranking taxonomy of contigs (e.g., bacterial contigs misannotated as *Mirusviricota*) and negatively affect the quality of our downstream analyses. To overcome this issue, we carefully decontaminated the database by performing an "all against all" diamond blast on its entire protein pool ("--ultra-sensitive" and percent identity of at least 50%. Since the best hit could be a contaminant, we worked on the 20 best hits per query (excluding the hit corresponding to the query) and quantified for each protein the proportion of hits (up to 19) corresponding to each of the high-ranking taxonomic categories. Most proteins

had an overwhelming signal for one high-ranking taxonomic category that aligned with the taxonomy of its corresponding genome (e.g., most proteins from bacterial MAGs only have signal for "Bacteria"). Since our goal was to identify contaminants at the contig level, we merged the protein-level signal at the level of individual contigs ("cumulative signal of all the proteins in the contig"), providing this time a contig-level proportion of hits corresponding to each of the high-ranking taxonomic categories. As expected, most contigs had an overwhelming signal for one high-ranking taxonomic category that aligned with the taxonomy of its corresponding genome. However, we could also detect clear contaminants: contigs with an overwhelming signal for one high-ranking taxonomic category that did not align with the taxonomy of its corresponding genome. We used different criteria, all based on the "all against all" diamond blast, to identify and remove contaminants from the genomic database:

- **For the non-viral high-rank taxonomic categories**, we removed contigs if less than 25% of the cumulative signal of all the proteins in the contig corresponded to the taxonomy of its corresponding genome. For example, a bacterial contig with 10 proteins that provided a total of 150 "top 20" best hits, 100 of which corresponding to the high-ranking taxonomy "Bacteria", would be labelled as Bacteria and kept in the database. But if only 10 of the 150 "top 20" best hits corresponded to the high-ranking taxonomy "Bacteria", the contig would have been labelled as a likely contaminant and removed.

- **For the two viral high-rank taxonomic categories (*Mirusviricota* and *Nucleocytoviricota*)**, we applied a more complex strategy due to the more complex evolutionary (high rate of genomic and protein sequence diversification) and ecological (notable level of gene flux with their eukaryotic hosts) properties. First, we selected contigs that met any of the following criteria: (1) at least 25% of the cumulative signal of all the proteins in the contig corresponds to the taxonomy of its corresponding genome, (2) the "hit ratio" (defined as the total number of "top 20" hits in the contig divided by the number of proteins in the contig) is equal or below 0.2, (3) or the percent of proteins in the contig with at least one hit is below 10%. The extremely low value cutoffs selected in the second and third criteria were chosen to identify contigs that are highly divergent from the rest of the genomic database, which we rarely observed among the non-viral high-rank taxonomic categories. For contigs that did not meet any of those three criteria, we removed contigs if cumulative signal of all its proteins reached at least 50% when excluding both the "Eukaryotes" (the viral host category) and the viral taxonomy of the corresponding genome (*Mirusviricota* or *Nucleocytoviricota*). Finally, we also removed contigs that did not have any hit among the cumulative signal of all its proteins for the viral taxonomy of the corresponding genome (*Mirusviricota* or *Nucleocytoviricota*). Note that all of those final criteria were defined after carefully exploring the "all against all" diamond blast results, and after manually testing a wide range of cutoffs.

In total, we removed 6,480 contigs (cumulative length of 34.8 Mb) out of the 6,157,614 contigs initially present in the database (cumulative length of 29.9 Gb). Note that ~5.7 million contigs correspond to large and highly fragmented eukaryotic genomes. The removal of just 0.1% of contigs (and 0.12% of the volume) emphasizes the overall quality of the manual binning outcomes from *Tara* Oceans over the course of ten years. Critically, the now curated reference database dramatically improved the quality of our iterative automated binning workflow and provides important opportunities to accurately estimate the high-ranking taxonomy of numerous metagenomic contigs far beyond the scope of our study.

High-ranking taxonomic inferences of mOTUs contigs during the first automated binning iteration: We performed a diamond blast using proteins from the 2,659 mOTUs bins as queries and proteins from the curated reference database as subject sequences (diamond blast v2.18 with "--ultra-sensitive" option and percent identity >=30%). We processed the high-ranking taxonomy of the best hit for each query (if any), and labelled contigs as *Mirusviricota* if they fit the two following criteria: (1) at least 25% of proteins in the contig have a best hit for a *Mirusviricota* protein, (2) and this percentage (the percentage of proteins with a best hit for a *Mirusviricota* protein) is above that of any other high-rank taxonomic category (Bacteria, Archaea, Eukarya, chloroplast, or Nucleocytoviricota). In addition, contigs labelled as *Mirusviricota* were also assigned to a *Mirusviricota* clade if at least 50% of the corresponding proteins had a best match for the same one. In this first automated binning iteration, a total of 57,513 contigs were labelled as *Mirusviricota* (cumulative length of 549.2 Mb). For each bin, contigs assigned to the same mirusvirus clade were subsequently assigned to a unique MAG ID, while those lacking clade-level taxonomic information were not considered (208 contigs; 1 Mb). From there, we excluded MAGs <50kb or >650kb in length. Aside from a few exceptions, no more than one MAG remained per bin. This first iteration allowed the automatic characterization of 1,993 *Mirusviricota* MAGs corresponding to 55,501 contigs (cumulative length of 546 Mb).

Improved genomic recoveries from a second automated binning iteration: We integrated proteins form the 1,993 newly characterized mirusvirus MAGs into the curated reference database vastly expanding the scope of *Mirusviricota* proteins) and performed a second automated binning iteration on the 2,659 mOTUs bins using the exact same guideline as done in the first iteration (see previous paragraph). Critically, this second iteration allowed the identification of 13,011 additional *Mirusviricota* contigs (gain of 83 Mb). In total, 71,320 contigs were labelled as *Mirusviricota* for a cumulative length of 640.5 Mb. With the now expanded scope of *Mirusviricota* contigs, we transitioned from 1,993 *Mirusviricota* MAGs (55,501 contigs - 546 Mb) in the first iteration to 2,142 *Mirusviricota* MAGs (68,512 contigs - 629.3 Mb) fitting the same criteria in the second iteration.

Identifying cryptic Mirusviricota contigs based on lack of taxonomic signal: At the end of iteration 2, we realized that many contigs containing a mirusvirus MCP were still not labelled as *Mirusviricota*, nor were they annotated to any of other high-ranking taxonomic category. In most cases, lack of high-ranking taxonomic

annotation was due to an unusually low percentage of proteins with a diamond blast hit, which prevented reaching the first criteria of our methodology (25% of proteins labelled as *Mirusviricota*). Among those cases, we found dozens of examples of mOTUs bins with a size compatible with an average mirusvirus genome length (between 200kb and 400kb). Interestingly, in those cases, we observed a low percentage of proteins with a diamond blast hit in all contigs and not just the one harbouring the mirusvirus MCP. Based on those observations, we hypothesized that many mirusvirus genomes evolutionary divergent from the ones we had already characterized occurred among the mOTUs bins but were thus far overlooked. To recover at least partially those distant viral genomes, we considered that "lack of taxonomic signal" in all the long contigs that were binned with a contig containing a mirusvirus MCP represented "the most relevant signal" to expand our genomic exploration of *Mirusviricota*. In other words, at that stage our main rational was that long contigs in bins with a mirusvirus MCP and displaying very low levels of diamond blast hits would most likely correspond to *Mirusviricota*. With that rational in mind, we collected contigs at the end of iteration 2 using the following strategy: we (1) excluded bins >1 Mbp, (2) excluded bins displaying a medium to high level of diamond blast hits (>25% of proteins with a hit), (3) excluded contigs <10kb in the remaining bins, (4) and finally excluded contigs with >10% of proteins having a best hit for any of the high-rank taxonomic categories of Bacteria, Archaea and chloroplasts. This provided a total of 3,771 contigs (cumulative length of 59 Mb) which we labelled as cryptic contigs. Following our guidelines, cryptic contigs were assigned to a unique MAG ID if their cumulative length reached at least 50 kb within the scope of the same mOTUs bin. This allowed the characterization of 251 cryptic MAGs >50 kb.

An original third iteration focused on cryptic *Mirusviricota* contigs: All the mirusvirus MAGs characterized from Iteration 2 (including the 251 cryptic MAGs; see previous section) were integrated into the curated reference database, which we used for a third iteration solely focused on expanding the scope of the cryptic MAGs. We used the same cut-offs as for the previous iterations and retained a total of 285 cryptic MAGs >50 kb in length (cumulative length of 72 Mb).

Combining MAGs from the three automated binning iterations: During the phase of merging MAGs from iterations 1 and 2, MAGs were put into two categories: (1) those related to *Demutovirales*, *Okeanovirales* and *Styxvirales* for which we could estimate a quality metric (completion minus redundancy) based on preliminary single copy core gene collections, (2) and those for which we did not have a quality metric. For the category 1, MAGs that increased in size during iteration 2 (due to the addition of one or multiple contigs) replaced the MAG from iteration 1 only if there was no decrease of the quality metric. For the category 2, MAGs that increased in size during iteration 2 always replaced the MAG from iteration 1. Finally, we added the 285 mirusvirus cryptic MAGs characterized after the third iteration. The database contained 2,427 MAGs for a cumulative length of 714.8 Mb (12,2% of the cumulative length of mOTUs bins). In the final non-redundant database of 1,257 mirusvirus genomes described in our study, 24 genomes correspond to MAGs from iteration 1, 819 genomes correspond to MAGs from iteration 2, and 195 genomes correspond to

cryptic MAGs from iteration 3. Those 195 cryptic MAGs represent most genomes (total of 257) corresponding to the 16 cryptic putative orders described in our study, stressing the importance of this last iteration to explore the evolutionary diversity of *Mirusviricota*.

- **Iterative automated binning of the SPIRE database:**

In addition to the mOTUs database, we also screened a smaller but still consequential number of metagenomic assemblies (n=16,801) from the SPIRE database[18]. Metagenomic assemblies selected from SPIRE were chosen so that they represented a wide range of environments, including freshwater ponds and lakes, marine habitats, soil and sediment samples, engineered systems such as wastewater treatment plants, and others. We only considered contigs >5 kb in length and performed a gene prediction with Prodigal v. 2.6.3 (-p meta option). We searched all predicted proteins against a collection of mirusvirus HMMs using hmmsearch (default parameters, bit score of 50 used for initial screening). These included HMMs reported previously for the MCP, portal, and triplex proteins[16], as well as a custom HMM made from family B DNA polymerase proteins of previously-reported mirusviruses[4]. Metagenomes that contained proteins with matches to these HMMs were then binned using MetaBat2 (parameter -s 50000)[12]. From there, we followed the same iterative automated binning workflow presented for the mOTUs bins. In the final non-redundant database of 1,257 mirusvirus genomes described in our study, 68 genomes correspond to MAGs (mostly from *Styxvirales*) characterized from the SPIRE database.

A series of single copy core gene collections for *Mirusviricota*: After performing preliminary phylogenetic analyses (based on the MCP) to define main evolutionary clades of *Mirusviricota* using all genomes available (including from manual binning and the iterative automated binning), we aimed at creating final clade-specific single copy core gene collections to estimate the completion and redundancy of mirusvirus genomes. We applied two simple rules: (1) a clade needed at least 10 genomes to be considered, (2) and there should be at least 20 single copy core genes in a collection. Thus, clades with limited number of genomes (under sampling issue) or without enough single copy core genes (diversity issue) were excluded. At the end, we successfully created 17 distinct collections of clade-specific single copy core genes (average of 55 genes per collection), that together, predicted the completion and redundancy of 933 out of the 1,257 MAGs. The average quality score (completion minus redundancy) of 84% for those 933 MAGs (with average redundancy of just 4,2%) is a strong argument supporting the overall quality of our database.

Biological relevance of the cryptic MAGs: As expected, most MAGs with no quality score estimation correspond to the cryptic putative orders characterized in the third iteration of our automated binning survey. The lack of this prominent quality metric for most cryptic MAGs is primarily due to under sampling of their corresponding lineages. We have pros and cons regarding the biological relevance of the cryptic MAGs. On the one side, cryptic MAGs should be viewed with relative caution due to two limits: lack of quality score and the occurrence of contigs with very low levels of

diamond blast hits for any of the high-ranking taxonomic categories (see section "*Identifying cryptic Mirusviricota contigs based on lack of taxonomic signal*"). On the other side, our characterization of several cryptic MAGs from the manual binning and curation (see example in Figure 1) demonstrates that those lineages are not an automated binning artifact, and more importantly, we identified hallmark genes of the virion module in many of the cryptic MAGs and their evolutionary signal was congruent. In conclusion, we are highly confident in the biological relevance of the cryptic MAGs, and the main issue remaining is the risk of intra-clade genomic redundancy. In the most striking example, the largest mirusvirus MAGs (MIRUS_G_0396 – 792 kb with 29 contigs) is affiliated with a cryptic putative order (singleton family), and we cannot rule out the possibility that the 29 contigs correspond to more than one cryptic genome.

Lessons learned about automated binning for mirusviruses: Among the 2.1 million contigs in 2,659 mOTUs bins, our workflow labelled 71,320 contigs as *Mirusviricota* (cumulative length of 640.5 Mb) during the first two iterations, and 5,817 additional contigs (cumulative length of 73.7 Mb) of them as cryptic *Mirusviricota* (unusual lack of high-ranking taxonomic signal) during the third iteration. Thus, *Mirusviricota* corresponds to just 12% of the volume of the mOTUs bins. The fact that a large majority of contigs in the mOTUs bins are not related to *Mirusviricota* stresses the importance of decontaminations steps when performing automated binning for large and giant eukaryotic viruses.

Multiple rooting strategy for the RED normalization

Our taxonomic framework for *Mirusviricota*, which follows recent guidelines from *Nucleocytoviricota*[19], applied normalized rank assignments on our main phylogenomic tree (concatenation of MCP, portal and terminase genes) using relative evolutionary divergence (RED) score values (<0.22 for putative orders, and <0.65 for putative families). Critically, rooting of the tree directly impacts the RED score values. In the case of *Nucleocytoviricota*, with two well defined classes (*Megaviricetes* and *Pokkesviricetes*), a single rooting separating the two classes was logically applied prior to computing the RED scores[19]. However, when the relevant rooting (from an evolutionary standpoint, ideally using a proper outgroup) is unknown, the best practice (as indicated by GTDB) is to use multiple rooting scenarios, all using deep-branching demarcations, and then average the RED score values for the taxonomic framework ("RED values used for rank normalization are averaged over multiple plausible rootings" – source: https://gtdb.ecogenomic.org/methods). In the case of *Mirusviricota*, we do not have a proper outgroup to use and as a result, we do not know the root position. Thus, we used all possible deep-branching demarcations (total of 11) in an effort to optimize the relevance of the RED score values. The 11 selected positions are displayed in the Figure 3.

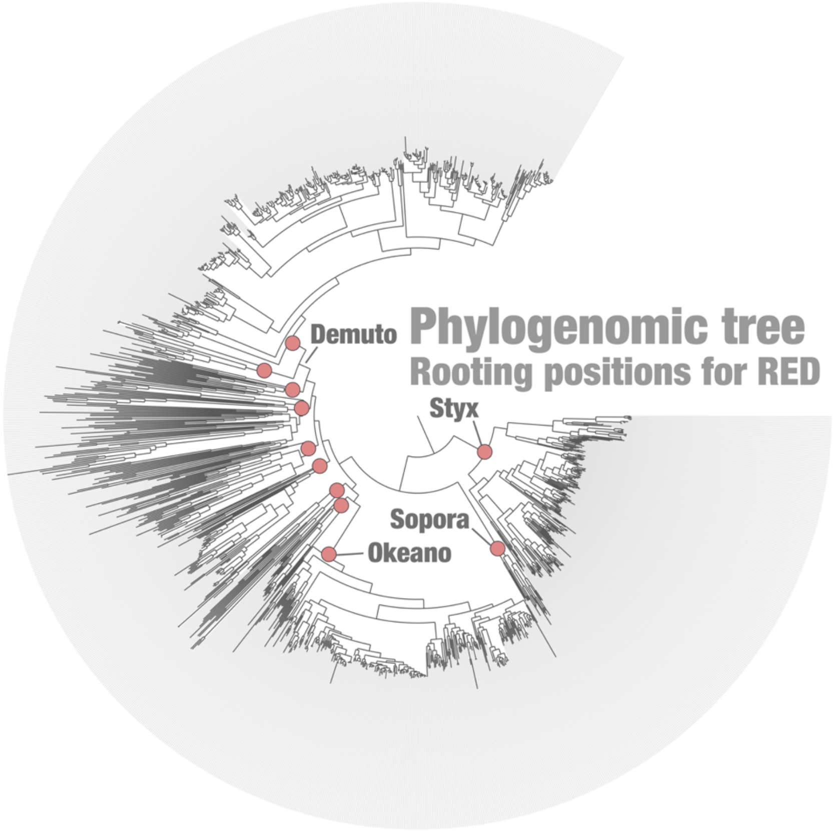

Figure 3: The 11 rooting positions for normalized rank assignments using relative evolutionary divergence**.**

Heliorhodopsins

Heliorhodopsins represent one of the rare core annotated functions shared by all major lineages of mirusviruses. Heliorhodopsins have already been identified as core functions in the first environmental genomic survey (restricted to *Demutovirales*) that focused on the sunlit oceans[4], and notably, were also identified in mirusviruses occurring in the aphotic layer of a lake[17]**.** While their exact role in the life cycle of mirusviruses remains highly elusive, their broad occurrence, including in orders predicted to infect the nucleus, stresses their importance for *Mirusviricota*.

References

1. Eren, A. M. *et al.* Anvi'o: an advanced analysis and visualization platform for 'omics data. *PeerJ* **3**, e1319 (2015).
2. Eren, A. M. *et al.* Community-led, integrated, reproducible multi-omics with anvi'o. *Nature Microbiology* Preprint at https://doi.org/10.1038/s41564-020-00834-3 (2021).

3. Delmont, T. O. *et al.* Functional repertoire convergence of distantly related eukaryotic plankton lineages abundant in the sunlit ocean. *Cell Genomics* 100123 (2022) doi:10.1016/J.XGEN.2022.100123.
4. Gaïa, M. *et al.* Mirusviruses link herpesviruses to giant viruses. *Nature 2023 616:7958* **616**, 783–789 (2023).
5. Jumper, J. *et al.* Highly accurate protein structure prediction with AlphaFold. *Nature 2021 596:7873* **596**, 583–589 (2021).
6. Fang, Y. *et al.* Genome-resolved year-round dynamics reveal a broad range of giant virus microdiversity. *mSystems* **10**, (2025).
7. Delmont, T. O. *et al.* Heterotrophic bacterial diazotrophs are more abundant than their cyanobacterial counterparts in metagenomes covering most of the sunlit ocean. *The ISME Journal 2021* 1–10 (2021) doi:10.1038/s41396-021-01135-1.
8. Delmont, T. O. *et al.* Nitrogen-fixing populations of Planctomycetes and Proteobacteria are abundant in surface ocean metagenomes. *Nature Microbiology 2018 3:7* **3**, 804–813 (2018).
9. Ruscheweyh, H. J. *et al.* Cultivation-independent genomes greatly expand taxonomic-profiling capabilities of mOTUs across various environments. *Microbiome* **10**, 1–12 (2022).
10. M, D. *et al.* The mOTUs online database provides web-accessible genomic context to taxonomic profiling of microbial communities. *Nucleic Acids Res* **1**, 13–14 (2024).
11. Nurk, S., Meleshko, D., Korobeynikov, A. & Pevzner, P. A. MetaSPAdes: A new versatile metagenomic assembler. *Genome Res* **27**, 824–834 (2017).
12. Kang, D. D. *et al.* MetaBAT 2: An adaptive binning algorithm for robust and efficient genome reconstruction from metagenome assemblies. *PeerJ* **2019**, (2019).
13. Hyatt, D. *et al.* Prodigal: prokaryotic gene recognition and translation initiation site identification. *BMC Bioinformatics* **11**, 119 (2010).
14. Jamy, M. *et al.* New deep-branching environmental plastid genomes on the algal tree of life. *bioRxiv* 2025.01.16.633336 (2025) doi:10.1101/2025.01.16.633336.
15. Collier, J. L. *et al.* The protist Aurantiochytrium has universal subtelomeric rDNAs and is a host for mirusviruses. *Current Biology* (2023) doi:10.1016/J.CUB.2023.10.009.
16. Zhao, H., Meng, L., Hikida, H. & Ogata, H. Eukaryotic genomic data uncover an extensive host range of mirusviruses. *Current Biology* **34**, 2633-2643.e3 (2024).
17. Zhang, L., Meng, L., Fang, Y., Ogata, H. & Okazaki, Y. Spatiotemporal dynamics of giant viruses within a deep freshwater lake reveal a distinct dark-water community. *ISME J* **18**, wrae182 (2024).
18. Schmidt, T. S. B. *et al.* SPIRE: a Searchable, Planetary-scale mIcrobiome REsource. *Nucleic Acids Res* **52**, D777–D783 (2024).
19. Aylward, F. O., Moniruzzaman, M., Ha, A. D. & Koonin, E. V. A phylogenomic framework for charting the diversity and evolution of giant viruses. *PLoS Biol* **19**, e3001430 (2021).
