# Supplementary material for: Widespread and intron-rich mirusviruses are predicted to reproduce in nuclei of unicellular eukaryotes
Source: Nat Microbiol. 2025 Nov 28;11(1):228–39. doi: 10.1038/s41564-025-02190-6 (PMC12768974; doi:10.1038/s41564-025-02190-6)
Supplement: Supplementary file 2 — Reporting Summary [file 41564_2025_2190_MOESM2_ESM.pdf]

# nature portfolio

Corresponding author(s): Tom O. Delmont

Last updated by author(s): Oct 2, 2025

# Reporting Summary

Nature Portfolio wishes to improve the reproducibility of the work that we publish. This form provides structure for consistency and transparency in reporting. For further information on Nature Portfolio policies, see our Editorial Policies and the Editorial Policy Checklist.

## Statistics

For all statistical analyses, confirm that the following items are present in the figure legend, table legend, main text, or Methods section.

| n/a | Confirmed | |
|---|---|---|
| ☐ | ☒ | The exact sample size (*n*) for each experimental group/condition, given as a discrete number and unit of measurement |
| ☐ | ☒ | A statement on whether measurements were taken from distinct samples or whether the same sample was measured repeatedly |
| ☐ | ☒ | The statistical test(s) used AND whether they are one- or two-sided<br>*Only common tests should be described solely by name; describe more complex techniques in the Methods section.* |
| ☒ | ☐ | A description of all covariates tested |
| ☒ | ☐ | A description of any assumptions or corrections, such as tests of normality and adjustment for multiple comparisons |
| ☒ | ☐ | A full description of the statistical parameters including central tendency (e.g. means) or other basic estimates (e.g. regression coefficient) AND variation (e.g. standard deviation) or associated estimates of uncertainty (e.g. confidence intervals) |
| ☐ | ☒ | For null hypothesis testing, the test statistic (e.g. *F*, *t*, *r*) with confidence intervals, effect sizes, degrees of freedom and *P* value noted<br>*Give P values as exact values whenever suitable.* |
| ☒ | ☐ | For Bayesian analysis, information on the choice of priors and Markov chain Monte Carlo settings |
| ☒ | ☐ | For hierarchical and complex designs, identification of the appropriate level for tests and full reporting of outcomes |
| ☐ | ☒ | Estimates of effect sizes (e.g. Cohen's *d*, Pearson's *r*), indicating how they were calculated |

*Our web collection on statistics for biologists contains articles on many of the points above.*

## Software and code

Policy information about availability of computer code

| | |
|---|---|
| Data collection | We collected publically available metagenomes. |
| Data analysis | anvi'o V.8 was used for data analysis. |

For manuscripts utilizing custom algorithms or software that are central to the research but not yet described in published literature, software must be made available to editors and reviewers. We strongly encourage code deposition in a community repository (e.g. GitHub). See the Nature Portfolio guidelines for submitting code & software for further information.

## Data

Policy information about availability of data

All manuscripts must include a data availability statement. This statement should provide the following information, where applicable:

- Accession codes, unique identifiers, or web links for publicly available datasets
- A description of any restrictions on data availability
- For clinical datasets or third party data, please ensure that the statement adheres to our policy

Data availability: All databases our study generated have been made publicly available at https://figshare.com/s/33ebaa0ef9edf2df1f83. Those include (1) MCP proteins identified in the global metagenomic survey, (2) the non-redundant database of 1,257 Mirusviricota genomes (both contigs and predicted genes in the form of nucleotide and protein coding sequences), (3) the intron-informed gene model for Mirusviricota, (4) HMMs for the Mirusviricota virion morphogenesis module, (5) the database of single-copy genomic MCPs used for the taxonomic annotations of metagenomic MCPs, (6) protein alignments and phylogenetic trees

corresponding to the MCP, portal and terminase hallmark genes, (7) intron sequences found in the genomic database, (8) the global protein database (Bacteria, Archaea, Eukarya, plastids, Nucleocytoviricota, Mirusviricota) used for high-ranking taxonomic annotation of contigs, (9) and our protein database for Nucleocytoviricota and Herpesvirales.

## Research involving human participants, their data, or biological material

Policy information about studies with human participants or human data. See also policy information about sex, gender (identity/presentation), and sexual orientation and race, ethnicity and racism.

| | |
|---|---|
| Reporting on sex and gender | No human participants |
| Reporting on race, ethnicity, or other socially relevant groupings | No human participants |
| Population characteristics | No human participants |
| Recruitment | No human participants |
| Ethics oversight | No human participants |

Note that full information on the approval of the study protocol must also be provided in the manuscript.

# Field-specific reporting

Please select the one below that is the best fit for your research. If you are not sure, read the appropriate sections before making your selection.

☐ Life sciences ☐ Behavioural & social sciences ☒ Ecological, evolutionary & environmental sciences

For a reference copy of the document with all sections, see nature.com/documents/nr-reporting-summary-flat.pdf

# Ecological, evolutionary & environmental sciences study design

All studies must disclose on these points even when the disclosure is negative.

| | |
|---|---|
| Study description | We solely explore metagenomic data. |
| Research sample | Our survey covers more than 100,000 metagenomic assemblies from a wide range of ecosystems. However, most of the signal for mirusviruses is aquatic. |
| Sampling strategy | No sampling was done |
| Data collection | No data collection. Only the use of available metagenomes. |
| Timing and spatial scale | No relevant |
| Data exclusions | No relevant |
| Reproducibility | No relevant |
| Randomization | No relevant |
| Blinding | No relevant |

Did the study involve field work? ☐ Yes ☒ No

# Reporting for specific materials, systems and methods

We require information from authors about some types of materials, experimental systems and methods used in many studies. Here, indicate whether each material, system or method listed is relevant to your study. If you are not sure if a list item applies to your research, read the appropriate section before selecting a response.

## Materials & experimental systems

| n/a | Involved in the study |
| --- | --- |
| ☒ | ☐ Antibodies |
| ☒ | ☐ Eukaryotic cell lines |
| ☒ | ☐ Palaeontology and archaeology |
| ☒ | ☐ Animals and other organisms |
| ☒ | ☐ Clinical data |
| ☒ | ☐ Dual use research of concern |
| ☒ | ☐ Plants |

## Methods

| n/a | Involved in the study |
| --- | --- |
| ☒ | ☐ ChIP-seq |
| ☒ | ☐ Flow cytometry |
| ☒ | ☐ MRI-based neuroimaging |

# Plants

| | |
| --- | --- |
| Seed stocks | No plants |
| Novel plant genotypes | No plants |
| Authentication | No plants |
